# Potential roles of stigma exsertion on spikelet fertility in rice (*Oryza sativa* L.) under heat stress

**DOI:** 10.3389/fpls.2022.983070

**Published:** 2022-09-21

**Authors:** Beibei Qi, Chao Wu

**Affiliations:** ^1^College of Agriculture, Guangxi University, Nanning, China; ^2^Guangxi Key Laboratory of Functional Phytochemicals Research and Utilization, Guangxi Institute of Botany, Guangxi Zhuang Autonomous Region and Chinese Academy of Sciences, Guilin, China

**Keywords:** rice, heat stress, spikelet fertility, flowering, stigma exsertion

## Abstract

Heat stress during the flowering stage induces declining spikelet fertility in rice plants, which is primarily attributed to poor pollination manifesting as insufficient pollen deposited on the stigma. Plant pollination is associated with anther dehiscence, pollen dispersal characteristics, and stigma morphology. The mechanisms underlying the responses of spikelet fertility to heat stress have been clarified in depth in terms of the morphological and behavioral characteristics of the male reproductive organs in rice. However, the roles of female reproductive organs, especially the stigma, on spikelet fertility under heat conditions are unclear. The present study reviews the superiority of stigma exsertion on pollen receptivity under heat during the flowering stage and discusses the variations in the effects of exserted stigma on alleviating injury under asymmetric heat (high daytime and high nighttime temperatures). The pollination advantages of exserted stigmas seem to be realized more under high nighttime temperatures than under high daytime temperatures. It is speculated that high stigma exsertion is beneficial to spikelet fertility under high nighttime temperatures but detrimental under high daytime temperatures. To cope with global warming, more attention should be given to rice stigma exsertion, which can be manipulated through QTL pyramiding and exogenous hormone application and has application potential to develop heat-tolerant rice varieties or innovate rice heat-resistant cultivation techniques, especially under high nighttime temperatures.

## Introduction

Global warming has caused rising surface temperatures and frequent extreme heat events. In the past 50 years, six large-scale extreme heat events have occurred in the middle and lower Yangtze River region (the main rice production area in China) during the midsummer season (Shi and Ye, 2021). Taking 2013 as an example, a heat event recorded a maximum temperature of >35°C for over 10 consecutive days that occurred in parts of Central China from July to August (Li et al., 2015), during which the flowering of midseason rice in the paddy occurs, and the extreme heat caused large-scale spikelet sterility and yield reduction (by 30 to 50% in some areas) (Guo et al., 2018). Notably, global warming shows the characteristics of asymmetric warming, with a higher temperature increase during the nighttime than during the daytime (Jiang et al., 2020). An analysis of historical data showed that rice grain yields have decreased by 10% for every 1°C increase in the minimum temperature at night (Peng et al., 2004). High nighttime temperature is thus regarded as an invisible natural disaster and has attracted great attention. Heat events have become the main agrometeorological disaster affecting rice production worldwide (Impa et al., 2021; Xu J. et al., 2021).

Previously, the effects of high temperatures on rice have mainly focused on high daytime temperatures during the reproductive stage (Fahad et al., 2017; Wu et al., 2019a). Unless otherwise specified, the “heat stress/treatment” mentioned in the present paper includes high daytime temperatures. Heat-induced reductions in spikelet fertility are attributed to the disturbance of the processes involved in pollination and fertilization (Matsui et al., 2021; Wang et al., 2021). Poor pollination manifests as insufficient pollen grains deposited on the stigma due to inhibited anther dehiscence and is the primary obstacle for reduced spikelet fertility induced by asymmetric heat (high daytime and high nighttime temperatures) (Song and Wu, 2020; Wu et al., 2020). Plant pollination refers to the process in which pollen is released from the anther and deposited on the stigmas. The mechanisms of heat effects on rice pollination and spikelet fertility have been investigated in terms of the response characteristics of male reproductive organs such as anthers and pollen (Fahad et al., 2018; Matsui et al., 2021). Even though the stigma is the receiver of pollen grains and the pollen germination site, the roles of female reproductive organs on pollination and spikelet fertility under heat have rarely been studied (Wu et al., 2019b; Xu Y. et al., 2021).

Stigma exsertion, which is a key determinant of the rice mating system among the stigma morphological indices, greatly contributes to pollination and has been widely utilized in hybrid rice seed production due to its pollination advantages (Tien et al., 2013). However, the effect of stigma exsertion on the pollination and spikelet fertility of rice under heat remains poorly understood (Wu et al., 2019a). The causes of the differences in rice varieties in response to high daytime and nighttime temperatures and the underlying mechanisms have long attracted the attention of investigators worldwide (Impa et al., 2021; Xu Y. et al., 2021); however, despite a focus on the detrimental effects of heat stress on rice, little progress has been made in this regard (Wu et al., 2019b). It is unclear (i) whether the advantages of pollination in exserted stigmas can compensate for an insufficient stigma pollen count induced by heat and mitigate heat injury on rice spikelet fertility during the flowering stage and (ii) whether the effects of stigma exsertion on spikelet fertility are different between high daytime temperature and high nighttime temperature given the possibility that the pollination advantages of exserted stigmas may be different during these exposures.

In the present review, the benefits of exserted stigma on pollen receptivity under heat stress during the flowering stage are analyzed, the different effects of exserted stigma on the alleviation of heat injury under asymmetric heat are discussed, and the possible underpinning mechanisms are also explored. Based on previous findings, some further thoughts on the future direction of rice cultivation and breeding for heat resistance/tolerance in terms of stigma exsertion are proposed, which will help provide new clues for overcoming pollination barriers and exploring the mechanisms of reduced spikelet fertility induced by asymmetric heat during the flowering stage in rice.

## The pollination barrier is the key issue in reduced spikelet fertility under heat stress

Spikelet fertility of rice is associated with pollination and fertilization, which include four main stages: (i) anther dehiscence, which proceeds through the sequential processes of thickening of the inner wall of the locule, swelling of pollen grains by water absorption, rupturing of the septum, and dehydration and cracking of the stomium (Hong et al., 2022); (ii) pollen dispersal, through which pollen grains are released from the anther pores (Fahad et al., 2015), with the amount of pollen grains released depending on the size and shape of the anther pores and the viscosity of the pollen grains (Santiago and Sharkey, 2019); (iii) pollen transport, in which pollen grains are dispersed and deposited onto the stigmas, and the pollen counts on the stigma depend on the distance and the relative positions between anther pores and the stigma (Liu S. J. et al., 2015), as well as the microclimate of the canopy (Yoshimoto et al., 2020); and (iv) pollen germination, pollen tube elongation, and embryo sac fertilization (Matsui et al., 2021).

To varying degrees, heat stress during the flowering stage inhibits anther dehiscence, hinders the timely dispersal of pollen grains, decreases the number of pollen grains deposited on stigmas, inhibits pollen germination (Wu et al., 2019a), impedes pollen tube elongation on the stigma, and reduces embryo sac fertilization (Shi et al., 2018), ultimately leading to reduced spikelet fertility. Notably, the insufficient pollen grains deposited on the stigma caused by unsmooth pollen shedding is the primary reason why heat stress reduces spikelet fertility in rice during the flowering stage (Matsui et al., 1997). To ensure successful pollination and fertilization under natural conditions, there should be more than 10 germinated pollen grains on a stigma, requiring the deposition of more than 20 pollen grains on the stigma (Sawada, 1974; Matsui, 2005). However, pollen germination is reduced by heat, so even greater pollen counts are required on the stigma to ensure the necessary number of germinated pollen grains under heat conditions. Therefore, increasing the number of pollen grains deposited on stigmas is essential for coping with the reduced spikelet fertility induced by heat during the flowering stage.

As the initial step of pollination, anther dehiscence is highly susceptible to heat stress and thus was suggested to be a selective marker for screening heat tolerance (Kobayashi et al., 2011). The well-known heat-tolerant rice genotype Nagina 22 exhibits good anther dehiscence characteristics (Wu et al., 2019a), which is the main reason for its ability to maintain stable spikelet fertility under heat stress during the flowering stage (Cheabu et al., 2018). The physiological mechanisms underlying the heat effects on anther dehiscence during the flowering stage have been extensively investigated, and the main findings have been that (i) heat stress induces abnormalities in the inner wall of the theca, septum, and stomium tissue (Matsui and Omasa, 2002); (ii) heat stress affects osmotic adjustment substances, such as sucrose, silicon, potassium, and calcium ions, which disrupt water metabolism and finally disturb the water absorption and dehydration of anthers (Yan et al., 2002; Firon et al., 2006); and (iii) heat stress affects anther dehiscence through phytohormonal regulation (Sakata et al., 2010). In practice, the screened heat tolerance indices involving anthers, i.e., a high percentage of dehisced thecae, long dehiscence at the base of the thecae, and uniform anther dehiscence (Matsui, 2005), are used to develop and screen ideal rice genotypes of heat tolerance. The proposed cultivation techniques, such as the application of silicon (Wu et al., 2014), exogenous indoleacetic acid (Sakata et al., 2010), and plant growth regulators (Fahad et al., 2016a), and intensified pollination and fertilization measures (Wu et al., 2020), which aim at relieving the heat injury on anther dehiscence and pollen shedding, help mitigate the heat injury on spikelet fertility during the flowering stage.

To summarize, the pollination barrier due to insufficient pollen shedding onto the stigma is the key issue underlying low spikelet fertility under heat stress during the flowering stage. Previously, the causes of heat effects on pollination and spikelet fertility in rice and the underlying mechanisms have been explored in depth in terms of anther dehiscence, based on which countermeasures have been proposed. However, given that the stigma serves as the receiver of pollen and the site of pollen germination, the effect of the morphological characteristics of the stigma on pollination and spikelet fertility under heat stress during the flowering stage is less considered.

## The pollination advantages of exserted stigmas

**FIGURE 1:**
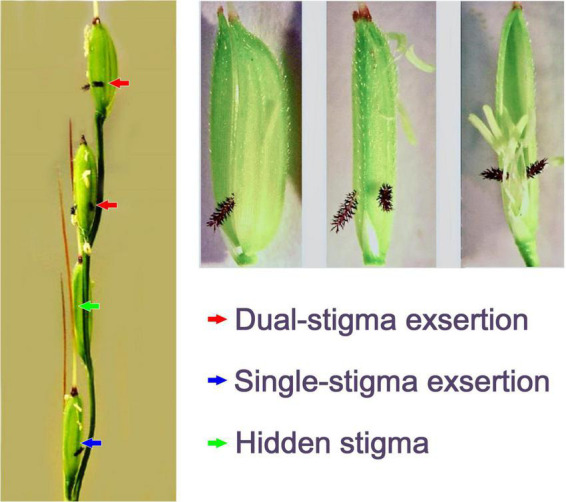
Illustration of stigma exsertion in rice [adapted from Yan et al. (2009) and Zhou et al. (2017) with some modifications].

Among the morphological and physiological characteristics of the stigma, stigma exsertion and stigma receptivity (vitality) play the most important roles in rice pollination and fertilization (Yu et al., 2006; Shivrain et al., 2007). Stigma exsertion refers to the phenomenon in which the stigma is exserted outside the palea and the lemma of the spikelet after floret opening in rice plants (Figure 1). It can be categorized into single-stigma exsertion, dual-stigma exsertion, and non-stigma exsertion (hidden stigma), depending on the number of stigmas exserted outside the spikelet (Akhilesh Singh et al., 2015). Spikelets with more exserted stigmas have the advantage of receiving more pollen grains, with the following ranking: spikelet with dual-stigma exsertion > spikelet with single-stigma exsertion > spikelet without exserted stigma (Longkumer and Deka, 2015). In hybrid rice seed production, cytoplasmic male-sterile rice lines with a high percentage of stigma exsertion are preferred to increase seed production by increasing the outcrossing rate due to the superiority of pollen receptivity of the exserted stigmas (Yan et al., 2009).

**FIGURE 2:**
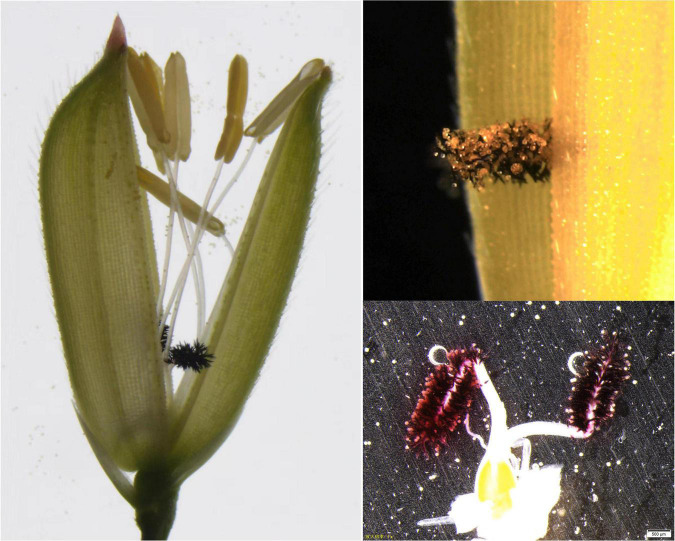
Illustration of the pollination advantage of the exserted stigma.

The pollination advantages of the exserted stigmas are manifested as follows. (i) Stigma exsertion enlarges the spatial basis of pollination. The exserted stigmas show a high degree of expansion (Figure 2) and a larger area for pollen receipt (Farrell et al., 2006; Molano-Flores and Faivre, 2014). (ii) Stigma exsertion prolongs the pollination period. The exserted stigma breaks through the physical barrier of the lemma and palea because it can capture pollen grains released at any time after flowering regardless of the non-synchronous flowering induced by heat during the flowering stage (Figure 2). (iii) Stigma exsertion may facilitate pollen germination by benefitting from the pollen population effect, which could be induced by increasing pollen density on the exserted stigma (Boavida and Mccormick, 2007; Zhang et al., 2010). In summary, stigma exsertion has the advantage of increasing the pollen counts on the stigma and improving the percentage of pollen germination.

**FIGURE 3:**
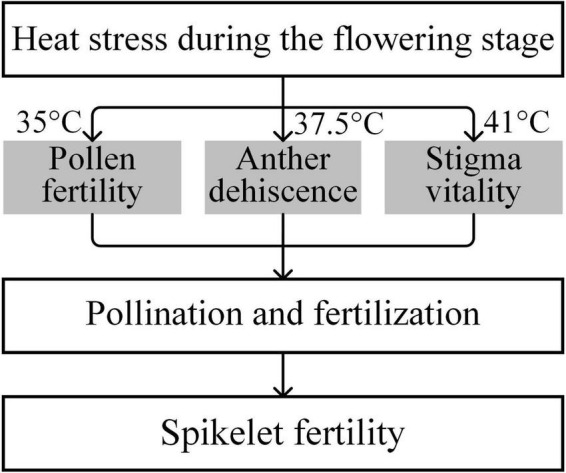
Critical temperatures of heat injury on pollen fertility, anther dehiscence, and stigma vitality (adapted from Wu, 2016).

Stigma receptivity influences the success of pollination in rice under heat conditions. The duration of stigma receptivity can last for 4–7 days in rice; the vitality of the exserted stigma remains normal during the 1–2 days after flowering, and the receptivity begins to decline on the third day and is completely lost on the seventh day after flowering (Xiao et al., 2015). In rice plants, the stigmas are more tolerant of heat than the male reproductive organs (Wu et al., 2019a). Generally, the threshold temperatures for pollen fertility, anther dehiscence, and stigma vitality of the rice variety were 35, 37.5, and 41°C, respectively (Figure 3). Similarly, Dupuis and Dumas (1990) observed completely aborted pollen, but receptivity of stigmas was still detected in maize when exposed to high temperature at 40°C. In cowpea, heat stress has relatively little effect on stigma vitality (Warrag and Hall, 1984). We previously observed that the receptivity of exserted stigmas was not significantly affected by heat treatments at 09:30 or 11:30 and did not significantly decrease until 16:00. It should be noted that the receptivity of exserted stigmas was still as high as 78.1% (73.3–82.5%) at 16:00 under heat conditions (Wu et al., 2019a). Evidently, the exserted stigmas of rice plants showed relatively stable vitality, which could give them a pollination advantage under heat stress when temperatures are below 41°C, the threshold temperature for stigma vitality (Figure 3), in either *japonica* or *indica* rice.

## Potential roles of stigma exsertion on pollination and spikelet fertility under high daytime temperature

High daytime temperature during the flowering stage delayed the dehiscence of anthers; thus, asynchronous anther dehiscence misses the flowering time, resulting in insufficient pollen grains deposited on the stigma. However, we observed that the uncracked anthers induced by heat treatment at flowering time could eventually dehisce and release pollen grains over time. After the lemma and palea have closed, the anthers usually remain outside the lemma and palea, while the stigmas are either exserted outside of or enclosed within the lemma and palea (Figures 1, 2). Under heat conditions, anther dehiscence and pollen dispersal are delayed so that they miss flowering time, the hidden stigmas cannot be pollinated at flowering, and there will never be another pollination opportunity due to the physical separation of the lemma and palea, finally resulting in reduced spikelet fertility in spikelets with hidden stigmas.

However, the exserted stigmas are not restricted to being pollinated by flowering time and can receive pollen grains at any time after flowering (Figure 2), and the delayed pollen dispersal induced by heat stress is no longer a challenge but, on the contrary, provides an opportunity for demonstrating the pollination advantages of exserted stigmas under heat conditions. Thus, the exserted stigma can (i) withstand heat stress and (ii) facilitate pollen germination induced by the pollen population effect under heat conditions during the flowering stage. Therefore, the exserted stigmas have a greater success of pollination than the hidden stigmas under heat conditions (Wu et al., 2019a). It is thought that the pollination advantage of the exserted stigma may help mitigate the adverse effects of heat stress on spikelet fertility at flowering.

The stigma exsertion and heat tolerance vary among rice subspecies (Table 1). The stigma exsertion of *indica* rice (20%) is higher than that of *japonica* rice (5.9%), and *indica* rice varieties are generally more heat-tolerant than *japonica* rice varieties (Matsui et al., 1997). However, the stigma exsertion of hybrid rice is generally higher than that of conventional rice, but hybrid rice varieties are usually more sensitive to heat stress than conventional rice varieties (Prasad et al., 2006). Therefore, the relationship between stigma exsertion and the heat tolerance of rice varieties is unclear. Previously, the low degree of stigma exsertion was assumed to be associated with high spikelet fertility in rice plants under heat conditions (Wassmann et al., 2008). Recently, a negative correlation between the percentage of stigma exsertion and spikelet fertility in rice genotypes was observed under high daytime temperature treatment (Wu et al., 2019a). In tomato cultivars, stigma exsertion was demonstrated to be unhelpful for screening high fruit set under heat stress (Lohar and Peat, 1998). Heat-induced stigma exsertion led to dyssynchrony of stamen-pistil, thus hindering pollination and resulting in lower fruit set in tomatoes (Pan et al., 2018). Previously, flowering time were reported to be partly associated with heat injury in rice varieties (Ishimaru et al., 2010), however, the dyssynchrony of stamen-pistil due to stigma exsertion under heat condition is independent of flowering time because it can be induced by heat whenever flowering occurs during the day. Taken together, these results suggest that high stigma exsertion may have a negative effect on spikelet fertility under high daytime temperatures, regardless of flowering time. The mechanisms of the effects of stigma exsertion on spikelet fertility in rice under heat stress merit in-depth investigation.

**TABLE 1:** The stigma exsertion and heat tolerance of rice subspecies.

Stigma exsertion	Heat tolerance
*Indica* > Japonica	*Indica* > Japonica
Hybrids > Inbreds	Hybrids < Inbreds

## Potential roles of stigma exsertion on spikelet fertility under high nighttime temperatures

Global warming has exhibited profound asymmetries, manifesting as a higher temperature increase during the nighttime than during the daytime (Peng et al., 2004). The optimum nighttime temperature for rice growth is 20–25°C (Owen, 1972), and serious spikelet sterility is induced when the nighttime temperature exceeds 30°C during the flowering stage (Jagadish et al., 2015). Although the spikelet fertility of rice responds differently to high daytime and nighttime temperatures (Coast et al., 2015; Song et al., 2021), high nighttime temperatures share the same mechanisms that induce lower spikelet fertility as high daytime temperatures, both of which hinder pollination by inhibiting anther dehiscence and impairing pollen germination (Mohammed and Tarpley, 2009; Fahad et al., 2016b). Therefore, the key issue to alleviating the injury to spikelet fertility caused by high nighttime temperature is still to increase the pollen counts deposited and germinated on the stigmas during the flowering stage.

The temperature during the daytime is the normal control under a high nighttime temperature treatment (Song et al., 2021; Wu et al., 2021), during which the receptivity of exserted stigmas and the pollination and fertilization of rice are almost unaffected by the nighttime temperature (Song, 2020). In cowpea (*Vigna unguiculata*), high nighttime temperature treatment negligibly impacted stigma receptivity (Warrag and Hall, 1984). Therefore, high nighttime temperature has less of an effect on the pollination advantages of the exserted stigma than high daytime temperature, and the pollination advantages of the exserted stigma can be more readily realized under high nighttime temperature. According to a recent observation, (i) rice genotypes with a higher degree of stigma exsertion couple with relatively higher spikelet fertility under high nighttime temperatures; (ii) increasing stigma exsertion by gibberellin A_3_ application augments spikelet fertility, and depressing stigma exsertion by paclobutrazol application reduces spikelet fertility, irrespective of the tolerance to heat of rice genotypes (Song, 2020). These observations indicate that high stigma exsertion may be beneficial for obtaining higher spikelet fertility under high nighttime temperatures.

## Conclusion and perspectives

An insufficient pollen count on the stigma is the primary factor underlying reduced spikelet fertility caused by heat stress during the flowering stage. Exserted stigmas have certain pollination advantages and can continuously receive pollen released by delayed dehiscent anthers caused by heat stress, thus having the potential to mitigate the injurious effect of heat stress on spikelet fertility. However, the roles of the exserted stigma on spikelet fertility are quite different under asymmetric heat. High stigma exsertion may be positive for obtaining higher spikelet fertility under high nighttime temperatures but may be negative for spikelet fertility under high daytime temperatures. Under heat conditions, particularly high nighttime temperatures, it is more feasible to develop heat-tolerant rice varieties or innovate rice heat-resistant cultivation techniques by modifying stigma exsertion than by restoring anther dehiscence. The great application potential of stigma exsertion is thus emphasized and deserves more attention during the course of combating heat stress under global warming.

Floral organ development can be manipulated by genetic modification (Gong and He, 2014). Stigma exsertion in the rice genotype is controlled by polygenes. To date, many closely linked QTLs controlling stigma exsertion have been identified (Table 2), parts of which have been used in QTL pyramiding for increasing seed production in hybrid rice. Interestingly, overexpression of *OsPID* can lead to the formation of extra stigmas in rice (He et al., 2019), and the pollination advantage would be amplified with three exogenous stigmas in rice varieties carrying *OsPID*. In addition, phytohormones significantly regulate stigma exsertion. It has been reported that *OsPID* regulates stigma exsertion through auxin signaling in rice plants (Xu M. et al., 2019). Gibberellins are required for development of floral organs in high plants (Li et al., 2021). It is common to increase the stigma exsertion of the female parent through gibberellin application to promote seed formation during the course of hybrid rice production (Du and Qi, 2015). Under heat conditions, the percentage of stigma exsertion can be increased by gibberellin A_3_ and jasmonate application and decreased by paclobutrazol application in rice and tomato plants (Wang et al., 2015; Pan et al., 2018; Wu et al., 2019a; Song, 2020).

**TABLE 2:** Genes controlling stigma exsertion in rice.

Genes	Trait	Interval/section	Source	References
*qPEST-5*; *qPEST-8*	Percentage of exserted stigma	*qPEST-5:* chromosome 5 (R2289∼R1553); *qPEST-8:* chromosome 8 (G1149∼G1963)	Dongxiang (common wild rice)	Li et al., 2001
*qPES-1a*; *qPES-1b*	Percentage of exserted stigma	*qPES-1a:* chromosome 1 (RM490∼RM259); *qPES-1b:* chromosome 1 (RM472∼RM104)	Zhenshan 97B (commercial cytoplasmic male sterility maintainer)	Yu et al., 2006
*qES3*	Percentage of exserted stigma	chromosome 3 (D83726 *Bst*Z17I∼T86)	IR24 (indica rice variety)	Miyata et al., 2007
*qPSE-2*; *qPES-5*; *qSPES-8*	Percentage of exserted stigma	*qPSE-2*: chromosome 2 (RM1285∼RM12595); *qPES-5*: chromosome 5 (RM17952∼RM18114); *qSPES-8*: chromosome 8 (RM8020∼RM7080)	You 1B (indica rice variety)	Deng et al., 2010
*GS3*	Stigma elongation	chromosome 3 (IND120∼HJ40)	IR24 (indica rice variety)	Takano-Kai et al., 2011
*qSe1*; *qSe6*	Percentage of exserted stigma	*qSe1*: chromosome 1 (RM10105∼RM283); *qSe6*: chromosome 2 (RM253∼RM4-1)	Chuanxiang 29A (superior rice cultivar)	Li et al., 2014
*qPSES5*; *qPDES-5*	Percentage of exserted stigma	*qPSES5:* chromosome 5 (RM3575∼RM3351); *qPDES-5:* chromosome 5 (RM3575∼RM3351)	K17B, Huhan1B (*indica* cytoplasmic male sterility maintainer)	Lou et al., 2014
*qSTL3*	Stigma length	19.8-kb region in the middle of the short arm of chromosome 3	Kasalath (*indicia* cultivar)	Liu Q. et al., 2015
*qSSE10*; *qSSE11*; *qDSE10*; *qDSE11*; *qTSE6*; *qTSE10*; *qTSE11*	Single-stigma exsertion; Dual-stigma exsertion	*qSSE10:* chromosome 10 (InD133); *qSSE11*: chromosome 11 (RM286); *qDSE10*: chromosome 10 (InD133); *qDSE11*: chromosome 11 (RM286); *qTSE6*: chromosome 6 (InD94); *qTSE10*: chromosome 10 (InD133); *qTSE11*: chromosome 11 (RM286)	XieqingzaoB (*O. sativa* sp. *indica* Kato)	Rahman et al., 2017a
*qSSE5*; *qTSE5*	Single-stigma exsertion; Dual-stigma exsertion	*qSSE5*: chromosome 5 (RM3638); *qTSE5*: chromosome 5 (RM3638)	XieqingzaoB (*O. sativa* sp. *indica* Kato)	Rahman et al., 2017a
*qSE11*	Percentage of exserted stigma	a 350.7-kb region located on chromosome 11 (InD144∼RM5704)	XieqingzaoB (*O. sativa* sp. *indica* Kato)	Rahman et al., 2017b
*GW2*; *GW5*		*GW2*: a 103-kb region on chromosome 3 (Tw35293∼RM266); *GW5*: a 21-kb genomic DNA region between CAPS markers Cw5 and Cw6 on chromosome 5	Teqing (*indica* rice variety), IR24 (*indica* rice variety)	Weng et al., 2008; Zhou et al., 2017; Zhang et al., 2020
*qPSES -3*; *qPDES -3*; *qPES -3*	Percentage of exserted stigma	qPSES -3: chromosome 3 (RM5488∼I-3-21); qPDES -3: chromosome 3 (I-3-18∼RM5488); qPES -3: chromosome 3 (RM5488∼I-3-21)	Gui2136S (*indica* rice)	Zhang H. et al., 2018
*qSE7*	Percentage of exserted stigma	a 1000-kb region located on chromosome 7 (RM5436∼RM5499)	XieqingzaoB (*O. sativa* sp. *indica* Kato)	Zhang K. et al., 2018
*qSER3*; *qSER8*	Percentage of exserted stigma	*qSER3*: chromosome 3 (C5-indel2400); *qSER8*: chromosome 8 (C5-indel6873)	W0120 (*O. rufipogon* accession)	Bakti and Tanaka, 2019
*qSER-7*	Percentage of exserted stigma	a 28.4-kb region on chromosome 7 (RM3859∼Indel4373)	II-32B (*indica* cytoplasmic male sterility maintainer)	Liu et al., 2019
*qSER-3.1*	Percentage of exserted stigma	a 3.9-Mb genomic region spanning 28.2∼32.1 Mb on chromosome 3	ZS616 (*indica* doubled haploid line)	Xu S. et al., 2019
*qSERb3-1*; *qSERb6-1*; *qSERb12-1*	Percentage of exserted stigma	*qSERb3-1*: chromosome 3 (PSM377∼RM487); *qSERb6-1*: chromosome 6 (RM589∼RM253); *qSERb12-1*: chromosome 12 (RM260∼RM309)	AA genome wild rice species	Zou et al., 2020
*qTSE3-1*; *qTSE3-2*; *qTSE6-2*; *qTSE11-1*	Percentage of exserted stigma	*qTSE3-1*: chromosome 3 (RM1350∼RM15466); *qTSE3-2*: chromosome 3 (RM1350∼RM15466); *qTSE6-2*: chromosome 6 (RM20615∼ RM19569); *qTSE11-1* on chromosome 11 (RM27183∼RM26213);	IR68897B (early duration maintainer line)	Gouri et al., 2021
*qSER-2a*; *qSER-2b*; *qSER-3a*; *qSER-3b*	Percentage of exserted stigma	*qSER-2a:* chromosome 2 (ID02M23∼RM3732); *qSER-2b:* chromosome 2 (ID02MQ51∼ID03M81); *qSER-3a:* chromosome 3 (ID61∼ID03M81); *qSER-3b:* chromosome 3 (ID03M141-16∼ID03Ma31)	AA-genome *Oryza* species	Tan et al., 2021
*qSER-5*; *qSER-1b*; *qSER-8b*; *qSER-3*	Percentage of exserted stigma	*qSER-5*: chromosome 5 (ID05M12∼ ID05M16); *qSER-1b:* chromosome 1 (ID01M92∼RM7318); *qSER-8b:* chromosome 8 (ID08M42∼ ID08M47); *qSER-3*: chromosome 3 (ID03Ma1-1∼ ID03Ma07)	*O. glaberrima*	Tan et al., 2022b
*qSER3a-sat*	Percentage of exserted stigma	chromosome 3 (PSM16∼ID03M59-6)	*O. glaberrima*	Tan et al., 2022a
*qSE4*	Percentage of exserted stigma	within 410.4 kb between markers RM17157∼RM17227 on chromosome 4	D50 (tropical japonica rice)	Guo et al., 2022
*qDSE1;qDSE8*	Percentage of exserted stigma	*qDSE1:* chromosome 1 (RM490∼RM259); *qDSE8*: chromosome 8 (RM152∼RM52)	Zhenshan 97B (commercial cytoplasmic male sterility maintainer)	Liu et al., 2022

Thus, stigma exsertion can be manipulated through molecular engineering approaches and/or the application of exogenous hormones, which have great potential to create heat-tolerant rice varieties. However, the degree of stigma exsertion should be manipulated appropriately to obtain full potential of spikelet fertility under the situation of high temperatures during the whole day because trade-off effects should be considered between high daytime temperature and high nighttime temperature in terms of the pollination advantage of exserted stigmas in rice.

## Author contributions

CW performed the experiments. BQ analyzed the data and compiled the figures. BQ and CW wrote the manuscript. CW edited the final manuscript. Both authors contributed to the article and approved the submitted version.
